# An Immune Feature-Based, Three-Gene Scoring System for Prognostic Prediction of Head-and-Neck Squamous Cell Carcinoma

**DOI:** 10.3389/fonc.2021.739182

**Published:** 2022-01-11

**Authors:** Yamin Zhang, Xiayan Luo, Jing Yu, Kejia Qian, Huiyong Zhu

**Affiliations:** ^1^ Department of Oral and Maxillofacial Surgery, The First Affiliated Hospital of Zhejiang University School of Medicine, Hangzhou, China; ^2^ School of Stomatology, Key Laboratory of Oral Biomedical Research of Zhejiang Province, Hangzhou, China

**Keywords:** HNSCC, lymph node metastasis, tumor-infiltrating lymphocyte, CD8+ T cell, LASSO, risk-scoring system

## Abstract

Head-and-neck squamous cell carcinoma (HNSCC) is characterized by a high frequency of neck lymph node metastasis (LNM), a key prognostic factor. Therefore, identifying the biological processes during LNM of HNSCC has significant clinical implications for risk stratification. This study performed Gene Ontology enrichment analysis of differentially expressed genes between tumors with LNM and those without LNM and identified the involvement of immune response in the lymphatic metastasis of HNSCC. We further identified greater infiltrations of CD8^+^ T cells in tumors than in adjacent normal tissues through immunochemistry in the patient cohort (*n* = 62), indicating the involvement of CD8^+^ T cells in the antitumor immunity. Hierarchical clustering analysis was conducted to initially identify the candidate genes relevant to lymphocyte-mediated antitumor response. The candidate genes were applied to construct a LASSO Cox regression analysis model. Three genes were eventually screened out as progression‐related differentially expressed candidates in HNSCC and a risk scoring system was established based on LASSO Cox regression model to predict the outcome in patients with HNSCC. The score was calculated using the formula: 0.0636 × CXCL11 − 0.4619 × CXCR3 + 0.2398 × CCR5. Patients with high scores had significantly worse overall survival than those with low scores (*p* < 0.001). The risk score showed good performance in characterizing tumor-infiltrating lymphocytes and provided a theoretical basis for stratifying patients receiving immune therapies. Additionally, a nomogram including the risk score, age, and TNM stage was constructed. The prediction model displayed marginally better discrimination ability and higher agreement in predicting the survival of patients with HNSCC compared with the TNM stage.

## 1 Introduction

Head-and-neck squamous cell carcinoma (HNSCC) is the most common malignant tumor arising from the head-and-neck cancers (1); it typically arises in the oral cavity, oropharynx, larynx, and hypopharynx (2, 3). HNSCC is characterized by frequent local invasiveness and neck lymph node metastasis (LNM), which have been identified as key prognostic factors. Despite advancements in diagnostic and therapeutic modalities, the prognosis of patients with HNSCC remains poor (4). Accurate prediction of prognosis assists in decision-making regarding adjuvant treatment after tumor resection. Currently, prognostic prediction and treatment decisions regarding HNSCC are based on the American Joint Committee on Cancer TNM staging system (5). However, the sensitivity and accuracy of this system for prognostic prediction are reduced by the individual heterogeneity (6). Accurate predictors are thus needed.

Innate and adaptive immunity can exert antitumor effect through recognition and elimination of malignant cells (7, 8). Increasing evidence suggests that densities of tumor-infiltrating lymphocytes (TILs) reflect the antitumor immunity process in the tumor environment and can predict overall survival (OS) of patients with cancer, including those with HNSCC (9–11). TILs comprise at least 28 different types (12), among which T lymphocytes are considered the central players (13). Besides T lymphocytes, various myeloid cells, such as dendritic cells (DCs), natural killer (NK) cells, and macrophages, also infiltrate the tumor microenvironment (TME) to exert their antitumor effects (14). Some studies have focused on the correlations between the infiltration of one or several immune cells in HNSCC tumors and prognosis (15–17). However, immunity in the tumor environment is mutually regulated by multiple TILs, which demands a comprehensive analysis of the TIL profiles.

Generally, TILs can effectively eliminate cancer cells at their early stages (8). However, cancer cells can evade the immune surveillance and resist the cytotoxic effect of cytotoxic T lymphocytes by hijacking immune-checkpoint pathways (18, 19), allowing the occurrence of advanced tumors. Multiple immune checkpoints, especially those expressed on the T-lymphocyte markers have been reported. Numerous clinical trials in various tumors have proven the efficiency of immune-checkpoint blockade (ICB) therapies, especially those that target cytotoxic T-lymphocyte-associated protein 4 (CTLA) and programmed cell death protein 1 (PD-1)/programmed cell death 1 ligand 1 (PD-L1) (20, 21). However, the clinical responses of ICB antibodies strongly depend on the composition of the TME (22, 23). As a result, the clinical benefit of patients with cancer from ICB therapies has great heterogeneity. A key challenge is the identification of patients potentially suitable for ICB therapies. Currently, the advancement of next-generation sequencing technologies and computational techniques allows analysis of the infiltration of immune cells (24–26). Based on the comprehensive insight of TIL profiles, we can better investigate the antitumor response and predict outcomes in patients with cancer. Some efficient risk scoring systems based on immune features have been reported in prediction of HNSCC outcome (27, 28). However, their clinical applications were somewhat limited by complex variables incorporated in prediction models.

In this study, we performed RNA sequencing in patients with HNSCC with different LNM statuses and found that the differentially expressed genes (DEGs) between HNSCC tumors with LNM and those without LNM were enriched in immune-response pathways. After estimating the abundance of TILs, we found that CD8^+^ T cells had greater estimated abundance in tumors with LNM, which was also verified in our HNSCC cohort by immunohistochemistry (IHC). Therefore, we focused on T-lymphocyte-related genes in the follow-up study. We developed a prognostic risk-scoring system based on the TIL-related genes, and the ability of the system in reflecting tumor immune environment was also evaluated.

## 2 Materials and Methods

### 2.1 RNA-Sequencing Profiles

#### 2.1.1 Sample Preparations and Procedures

Samples from LNM^−^ primary tumors (*n* = 4) and LNM^+^ primary tumors (*n* = 5) were cut into small specimens. The total RNA was extracted using Trizol reagent (Invitrogen, Carlsbad, CA, USA) following the manufacturer’s procedure. The purity and quantity of total RNA were analyzed using NanoDrop ND 1000 (NanoDrop, Wilmington, DE, USA), and the integrity of the RNA was assessed using Agilent 2100 with RIN number >7.0. Poly(A) RNA was purified from total RNA (5 µg) using poly T oligo attached magnetic beads using two rounds of purification (Invitrogen). The mRNA was then fragmented into small pieces using divalent cations under elevated temperature. Subsequently, the cleaved RNA fragments were reverse-transcribed to create the final cDNA library in accordance with the protocol for the mRNA-sequencing sample preparation kit (Illumina, San Diego, CA, USA). Lastly, we performed the 150-bp paired-end sequencing on an Illumina X Ten (LC Bio, Hangzhou, China) following the recommended protocols.

#### 2.1.2 Data Processing

The HISAT package (version 2.0.4) (29) was used to align the raw RNA sequences to the hg19 human reference genome (http://genome.ucsc.edu/). The mapped reads were assembled using StringTie (version 1.3.4) (30), and transcriptomes were merged using Perl scripts. The expression level for mRNAs was calculated by exon per million mapped reads (FRKM) using StringTie. The DEGs with |log2 (fold change)| >1 and *p*-value <0.05 were selected using “edgeR” (version 3.20.9) (31).

#### 2.1.3 Data Analyses

Gene Oncology (GO) enrichment was performed for DEGs using the GO database (http://geneontology.org/). The DEGs with significant differential expression (*p* < 0.01, log2|FC|>2) in top 20 enriched GO terms were selected and applied to GO enrichment analysis of ImmunoSystem Process using Cytoscape 3.1.0 (32). KEGG pathway analysis of the DEGs was also performed using the KEGG pathway database (https://www.genome.jp/kegg/pathway.html). Cell-type Identification by Estimating Relative Subsets of RNA Transcripts (CIBERSORT) (http://cibersort.stanford.edu) was used for TIL profiles. The algorithm was run using the leukocyte matrix (LM22) signature and 1,000 permutations for the estimation of relative fractions of multiple TILs in gene expression profiles of admixtures (26, 33, 34). Samples with statistically significant deconvolution result across all cell subsets (*p*-value <0.05) were included in the consequent analysis. The relative fractions of 22 TILs were summarized by means ± standard errors of the means (SEM).

### 2.2 IHC

#### 2.2.1 Sample Preparations and Procedures

A patient cohort (*n* = 62) with a histopathological diagnosis of primary HNSCC was enrolled in this study. All patients underwent surgical tumor resection and neck lymph node dissection (elective or therapeutic neck dissection) under general anesthesia at the First Affiliated Hospital of Zhejiang University from January 2018 to June 2021. The exclusion criteria were as follows: (i) chemotherapy, radiotherapy, or biological treatment before surgery; (ii) immune deficiency or immune system disease; (iii) inadequate clinicopathological medical records; (iv) previous history of other malignant tumors; and (v) previous history of primary tumors arising from the head and neck.

Demographical and clinicopathological data—sex, age, primary region of tumorigenesis, tumor size, and N status—were collected by a retrospective review of medical records and postoperative pathological reports. T and N staging was performed using the TNM staging system of the American Joint Committee on Cancer, 7th Edition. Because of the retrospective study design, power calculation was not performed. The sample size was equal to the number of patients treated in our institution during the recruitment period. The collection and the preservation of the samples were approved by the Ethics Committee of the First Affiliated Hospital, College of Medicine, Zhejiang University, and written informed consent was obtained from all participants.

Segments of tumor tissues (*n* = 62) and adjacent normal tissues (*n* = 24) (mucosa 5 cm beyond the edge of the carcinoma) were collected and repeatedly washed in phosphate-buffered saline (pH 7.4) to remove mucus and blood and then fixed in formalin, dehydrated, embedded in paraffin, and sectioned. Moreover, 4-μm-thick paraffin sections of samples were deparaffinized in xylene, rehydrated through graded alcohols, repaired with antigen retrieval through hot citric acid buffer (pH 6), and blocked with 3% bovine serum albumin. These sections were then incubated with antibody-CD4 (NCL-L-CD4-1F6, Leica Biosystems, Milton Keynes, UK), antibody-CD8 (NCL-L-CD8-4B11, Leica Biosystems, UK), antibody-Foxp3 (ab20034, Abcam, Cambridge, MA, USA), and secondary antibodies (Servicebio, Wuhan, China) successively at an appropriate dilution. Finally, sections were treated with 3,3′-diaminobenzidine, counterstained with hematoxylin, dehydrated through graded ethanol, cleared in xylene, and mounted with resin mounting medium.

#### 2.2.2 Quantitative Evaluation of Immunostaining Density

Immunostaining reactions were separately assessed by two independent pathologists who were blinded to the clinical data of the patients. A positive reaction was defined as clear brown staining. The interface of the tumor/normal tissues were screened at a low-power field (×100). Subsequently, areas containing the highest number of positively stained cells (hot spots) were selected. In the selected field, 3–8 separate areas of intense cells were captured in a ×200 field. These images were captured using an inverted microscope (Leica, Wetzlar, Germany).

We used the IHC Profile plugged in ImageJ software (USA) to semiautomatically calculate the intensity of positive cytoplasmic membrane staining and percentage of positive staining area. Antigen-expressed cells in each IHC image were divided into four levels (high positive, positive, low-positive, and negative) according to their density and assigned values of 3, 2, 1, and 0, respectively. We multiplied the value of positive cells and percentage of positive areas to obtain the IHC score of each marker based on the Barnes’ score method.

#### 2.2.3 Associations of TILs With Clinicopathological Features

Patients were divided into high- and low-infiltration groups based on their IHC scores of immune markers (CD8, CD4, and Foxp3). The Chi-squared test was performed to determine the associations of the immune markers with the clinicopathological features of patients with HNSCC.

### 2.3 Database Mining

#### 2.3.1 Data Acquisition

We mined The Cancer Genome Atlas (TCGA) database (https://portal.gdc.cancer.gov/) to extract the transcriptome data, pathological stage, and survival status of patients with HNSCC diagnosed between 1993 and 2013 (*n* = 501).

#### 2.3.2 Data Analyses

##### 2.3.2.1 Correlations of TIL-Related Genes by Pearson’s Analysis and Hierarchical Clustering Analysis

Pearson’s analysis was performed to screen DEGs closely related to CD8, CD4, and Foxp3, which were involved in the pathway of activation, differentiation, and migration of T cells. The correlation values were clustered and visualized through hierarchical clustering analysis (HCA) in the R software.

##### 2.3.2.2 Survival-Related Hub Gene Screening Using the LASSO Cox Regression Analysis

To identify the hub gene signatures relevant to survival of patients, we used a linear regression technique based on the LASSO algorithm in “glmnet” R (version 4.1-1). The most suitable signatures were selected by the LASSO Cox regression model when the minimum penalization coefficient (lambda) was obtained after running crossvalidation likelihood 1,000 times. The selected gene signatures were then applied to establish a risk-scoring system, by weighting the expression levels of gene signatures and corresponding regression coefficients. To validate its efficiency in predicting patients’ prognosis, patients with HNSCC were divided into the low- and high-risk groups based on their risk scores (median risk score as cutoff point). Survival rates of the two groups were calculated using the Kaplan–Meier (KM) method and compared using log-rank test. The time-dependent receiver operating characteristic curve (ROC) analysis was performed to assess the area under curve (AUC) for the 1‐, 3‐, and 5‐year OS, thus checking the survival prediction accuracy of the prognostic model.

##### 2.3.2.3 Survival Analysis

The correlations between the survival-related genes and OS of patients with HNSCC were analyzed using KM plotter database (http://kmplot.com/analysis/) (35). Cox proportional hazards regression analysis was performed to calculate the log-rank *p*-values, hazard ratios (HRs), and 95% confidence intervals (CIs). KM survival plots were generated to visualize the survival differences in patients with different mRNA expression levels of target genes (median as cutoff point).

##### 2.3.2.4 Associations Between the Risk Scores and Tumor Immune Microenvironment

The abundance of TILs in HNSCC tumors was estimated using the CIBERSORT algorithm. Tumors were divided into the high-risk and low-risk group (median as cutoff point). The estimated infiltration fractions of TILs between the two groups were compared using the Mann–Whitney *U* test. We also investigated the associations of the risk scores with the infiltrations of lymphoid and myeloid cells, respectively, using Pearson’s correlation test. Comparison of the overall infiltrations of lymphoid and myeloid cells between the two groups was also performed using the Mann–Whitney *U* test. Additionally, the associations of the risk scores with a set of immune checkpoints were analyzed using Pearson’s correlation test.

### 2.4 Quantitative Real-Time Polymerase Chain Reaction

The relative expression levels of the survival-related hub genes were further identified in LNM^−^ (*n* = 18) and LNM^+^ primary tumors (*n* = 18). Total RNA was isolated from collected tumor tissues using RnaExTM Total RNA Isolation Solution (GK3006, GENEray, Shanghai, China). Moreover, 1 μg of total RNA was used to synthesize cDNA. The quantitative real-time polymerase chain reaction (qRT‐PCR) was performed using 500 ng cDNA per 10 μl reaction. Each reaction was conducted with iQTM SYBR® Green Supermix (Bio‐Rad, Hercules, CA, USA). Gene amplification was conducted on thermal cycler programmed as follows: initial denaturation at 95°C for 5 min followed by 35 cycles at 95°C for 10 s, annealing at 60°C for 20 s, 72°C for 1 min, extending at 72°C for 5 min. Each sample was analyzed in triplicate. Relative expression levels were normalized to glyceraldehyde-3-phosphate dehydrogenase (GAPDH). The relative expression of targets in LNM^+^ tumors compared with LNM^−^ tumors was calculated using 2^−△△ct^. The primer sequences are presented in **Table 1**.

**Table 1:** Primers used in qRT-PCR.

Primer	Sequence
CXCL10_F	TATTTCCCTCACCTTTCCC
CXCL10_R	GCAGATTTGATTGCATACCTT
CXCL11_F	GAAAGGTGGGTGAAAGGAC
CXCL11_R	TGCAACAAGTAAGAACGTGAA
CCR5_F	TGTTTGCGTCTCTCCCA
CCR5_R	CCAGCCCCAAGATGACTA
GAPDH_F	CCTTCCGTGTCCCCACT
GAPDH_R	GCCTGCTTCACCACCTTC

### 2.5 Univariate and Multivariate Cox Regression Analyses

A total of 439 patients with complete clinical data from the TCGA dataset were evaluated, and univariate and multivariate Cox regression analyses were employed to investigate whether the risk score was an independent risk factor for the OS of patients with HNSCC. The OS rates were calculated using the KM method and log-rank test. We included age, grade, T stage, N stage, and TNM stage into the univariate Cox model, considering their potential prognostic roles. The risk score was classified into four levels by quartiles (low, low-medium, medium-high, and high); age of patients was classified into four age bands (<50, 50–60, 60–70, ≥70 years). Variables showing statistically significant effect (*p*-value <0.05) in the univariate analysis were included in the multivariate Cox regression model. Variables with *p*-values <0.05 in the multivariate Cox model were considered independent prognostic factors. The forest was used to display the HR, *p*-value, and 95% CI of each variable using the “forestplot” R package.

### 2.6 Construction and Assessment of the Nomogram Model

For convenient application of the established risk-scoring system in clinical work, we established a nomogram prediction model based on the risk scores and clinical parameters to predict outcomes of patients with HNSCC. Variables identified as independent risk factors were included to construct a nomogram prediction model to predict OS of patients with HNSCC. The discrimination of the constructed nomogram model was measured and compared using Harrell’s concordance index (c-index). The predicted accuracy of the nomogram for prediction of 1‐, 3‐, and 5‐year survival of patients with HNSCC was shown in the calibration curves and compared with that of the TNM stage (36).

### 2.7 Statistical Analysis

All statistical analyses and plots were conducted using GraphPad Prism (version 8.0) and R software (version 4.0.5). Student’s *t*-test was used for groupwise comparisons of normally distributed continuous variables; the Mann–Whitney *U* test was used for groupwise comparisons of variables with abnormal distributions. The Chi-squared test was used to analyze the associations between the TILs and clinicopathological features. Pearson’s correlation test was used to analyze correlations between groups. Correlation values were used to conduct HCA. The KM method was used to calculate survival rates. LASSO regression analysis was performed to filter key genes and establish the risk-scoring system. The accuracies of the diagnostic and prognostic prediction models were generated using ROC curves and calculated using the AUC. Univariate and multivariate Cox hazard regression analyses were performed for screening independent risk factors for the OS of patients with HNSCC. A nomogram was constructed based on parameters selected by multivariate Cox regression analysis. The discrimination abilities of the prognostic models were measured using the c-index. All statistical tests were two sided, and *p*-value <0.05 was considered statistically significant.

## 3 Results

### 3.1 Identification of Biological Processes in Tumors During LNM of HNSCC Tumors

**Figure 1:**
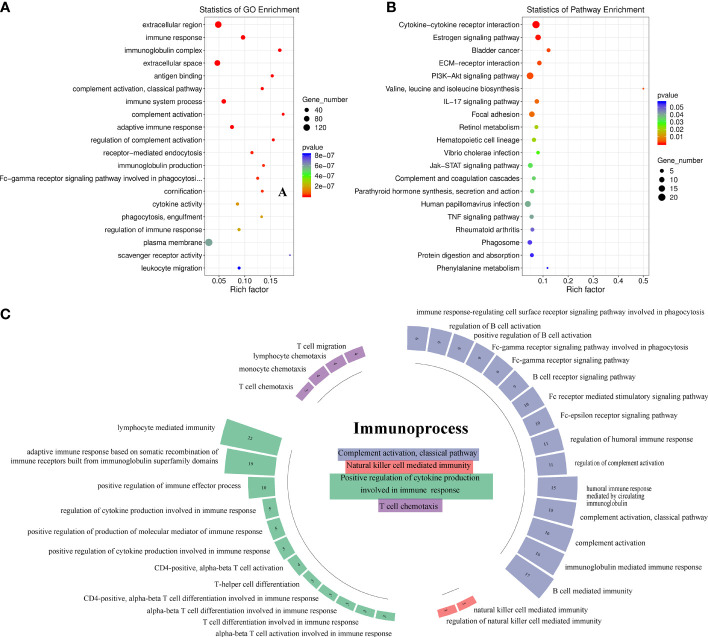
Identification of the immune process involved in antitumor lymphatic metastasis response in head-and-neck squamous cell carcinoma patients (*n* = 9). **(A)** Gene Ontology (GO) enrichment analysis of 523 differentially expressed gene (DEGs). The top 20 GO terms with the smallest *p*-value in the enrichment analysis results are presented. **(B)** KEGG pathway analysis of 523 DEGs. The top 20 pathways with the highest enrichment factor are shown. **(C)** Immunosystem process pathway enrichment analysis of 198 DEGs involved in the top 20 GO terms. Numbers in boxes indicate numbers of genes involved in corresponding immune pathways.

A total of 258 upregulated genes and 265 downregulated genes were identified in tumors with LNM compared with tumors without LNM (|log2 fold-change| >1 and *p*-value <0.05). DEGs were significantly enriched in extracellular matrix and immune-related GO terms (*p* < 0.01) (**Figure 1A**). Similarly, KEGG pathway analysis yielded DEGs enriched mostly in the cytokine-cytokine receptor interaction pathway (**Figure 1B**). We found the 198 DEGs enriched in the top 20 GO terms were mainly enriched in the immune system process pathways of complement activation (classical pathway) (53.85%), positive regulation of cytokines involved in immunity (34.63%), NK-mediated immunity (7.69%), and T-cell chemotaxis (3.85%) (**Figure 1C**). Among them, CCL26, MYB, CDH26, GATA3, CXCL10, CXCL11, IL6, and CCL20 were involved in the pathway of activation, differentiation, and migration of T cells.

### 3.2 Identification of Infiltrated TILs in HNSCC Tumors

**Figure 2:**
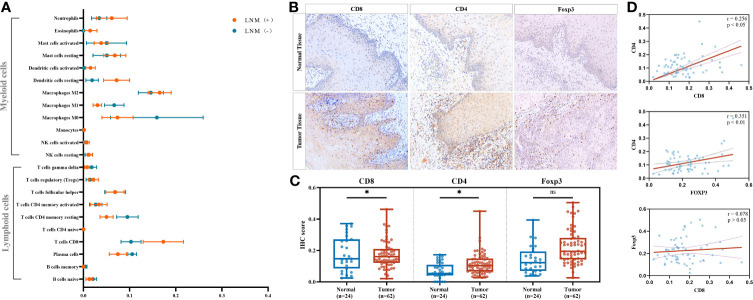
Identifying significant tumor-infiltrating lymphocyte (TIL) subtypes engaging in antitumor lymphatic metastasis response. **(A)** Comparison of the estimated fractions of 22 TILs in tumors with LNM (*n* = 5) and those without LNM (*n* = 4). All values were represented by mean ± SEM. **(B)** Representative immunohistochemistry (IHC) images of CD8, CD4, and FOXP3 in adjacent normal tissues and tumor tissues. Images were obtained under a light microscope at magnifications of ×200. **(C)** Comparison of the IHC scores between normal tissues (*n* = 24) and tumor tissues (*n* = 62) by Mann–Whitney *U* test. ^*^ *p* < 0.05; ns, *p* > 0.05. **(D)** Correlations of the IHC scores of CD8, CD4, and FOXP3 in tumor tissues (*n* = 62) by Pearson’s correlation test. TIL, tumor-infiltrating lymphocytes; LNM, lymph-node metastasis; SEM, standard error of mean.

We compared the average infiltration levels of TILs and found that CD8^+^ T cells were greater in LNM^+^ tumors than in LNM^−^ tumors (0.173 ± 0.044 versus 0.103 ± 0.022, respectively) (**Figure 2A**). IHC confirmed the expression of CD8 in tumor tissues and adjacent normal tissues. CD8^+^ T cells infiltrated the tumor stroma, invasive margin, and center, whereas CD4^+^ and Foxp3^+^ T cells mainly infiltrated the tumor stroma and invasive margin (**Figure 2B**). The IHC scores of CD8 and CD4 were significantly higher in tumor tissues (*p* < 0.05) while those of Foxp3 showed no significant difference (*p* > 0.05) (**Figure 2C**). The protein expression levels of CD8 and CD4 were significantly correlated (*r* = 0.256, *p* < 0.05), whereas CD8 and Foxp3 showed no statistically significant correlation (*r* = 0.078, *p* > 0.05) (**Figure 2D**). Additionally, the protein expression level of Foxp3 was highly relevant to that of CD4 (*r* = 0.351, *p* < 0.01). As shown in **Table 2**, the CD8 expression level was significantly associated with LNM status (*p* = 0.001) but not with sex, age, tumor site, tumor burden, or degree of differentiation. CD4 and Foxp3 showed no significant correlations with clinicopathological characteristics (*p* > 0.05).

**Table 2:** The Chi-square test of the associations between IHC scores and clinicopathological characteristics.

Variables	Cases	CD8			CD4			FOXP3		
		Low (%)	High (%)	*p*-value	Low (%)	High (%)	*p*-value	Low (%)	High (%)	*p*-value
**Gender**										
**Male**	34	15 (44.1%)	19 (55.9%)	0.307	15 (44.1%)	19 (55.9%)	0.307	16 (47.1%)	18 (52.9%)	0.61
**Female**	28	16 (57.1%)	12 (42.9%)		16 (57.1%)	12 (42.9%)		15 (53.6%)	13 (46.4%)	
**Age**										
**≤Median**	32	17 (53.1%)	15 (46.9%)	0.611	17 (53.1%)	15 (46.9%)	0.611	16 (50.0%)	16 (50.0%)	1
**>Median**	30	14 (46.7%)	16 (53.3%)		14 (46.7%)	16 (53.3%)		14 (46.7%)	16 (53.3%)	
**Region**										
**Gingiva**	16	9 (56.3%)	7 (43.8%)	0.501	7 (43.8%)	9 (56.3%)	0.779	6 (37.5%)	10 (62.5%)	0.368
**Tongue**	30	16 (53.3%)	14 (46.7%)		15 (50.0%)	15 (50%)		15 (50.0%)	15 (50%)	
**Others**	16	6 (37.5%)	10 (62.5%)		9 (56.3%)	7 (43.8%)		10 (62.5%)	6 (37.5%)	
**Tumor size**										
**≤2 cm**	23	13 (56.5%)	10 (43.5%)	0.43	13 (56.5%)	10 (43.5%)	0.43	11 (47.8%)	12 (52.2%)	0.793
**>2 cm**	39	18 (46.2%)	21 (53.8%)		18 (46.2%)	21 (53.8%)		20 (51.3%)	19 (48.7%)	
**Differentiation degree**										
**Well**	28	17 (60.7%)	11 (39.3%)	0.126	13 (46.4%)	15 (53.6%)	0.61	14 (50 %)	14 (50 %)	1
**Moderately/Poorly**	34	14 (41.2%)	20 (58.8%)		18 (52.9%)	16 (47.1%)		17 (50 %)	17 (50 %)	
**LNM**										
**LNM (−)**	35	24 (68.6%)	11 (31.4%)	**0.001**	18 (51.4%)	17 (48.6%)	0.798	17 (48.6%)	18 (51.4%)	0.798
**LNM (+)**	27	7 (25.9%)	20 (74.1%)		13 (48.1%)	14 (51.9%)		14 (51.9%)	13 (48.1%)	

LNM, lymph node metastasis; LNM(−), absence of lymph node metastasis; LNM(+), presence of lymph node metastasis.

The numbers in bold indicate statistical significance.

### 3.3 Identification of TIL-Related Genes

**Figure 3:**
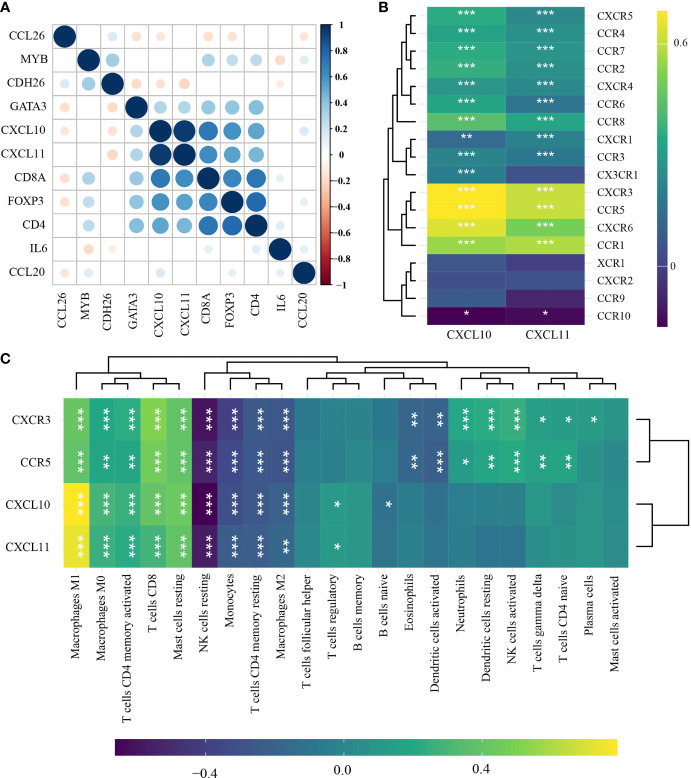
Identification of TIL-related genes in head-and-neck squamous cell carcinoma (*n* = 501) by Pearson’s correlation test and hierarchical cluster analysis. **(A)** Correlations among immune-related differentially expressed gene (DEGs), CD8A, CD4, and Foxp3. **(B)** Correlations of CXCL10 and CXCL11 with chemokine ligands. **(C)** Correlations of CXCL10, CXCL11, CXCR3, and CCR5 with the abundance of TILs. ^*^ *p* < 0.05; ^**^ *p* < 0.01; ^***^ *p* < 0.001. TILs, tumor-infiltrating lymphocytes.

Among the DEGs involved in T-cell regulation, CXCL10 and CXCL11 were identified to be highly correlated with TIL-characterizing gene sets (CD8A, CD4, and Foxp3) (*p* < 0.00001) (**Figure 3A**). Additionally, CXCR3 and CCR5 were highly correlated with both CXCL10 and CXCL11 (*r* > 0.613, *p* < 0.0001) (**Figure 3B**). Consistently, the chemokines were positively associated with a series of effector immune cells, including M2 macrophages, resting NK cells, resting mast cells, CD8^+^ T cells, and activated memory CD4^+^ T cells (TAM CD4) (*r* > 0.04, *p* < 0.001) (**Figure 3C**). Additionally, they were negatively associated with M1 macrophages, activated mast cells, plasma cells, and naïve CD4^+^ T cells (**Figure 3C**). Comprehensively, CXCL10, CXCL11, CXCR3, and CCR5 were TIL-related chemokines, involved in the accumulation of TILs in HNSCC.

### 3.4 Establishment and Validation of the Risk-Scoring System

**Figure 4:**
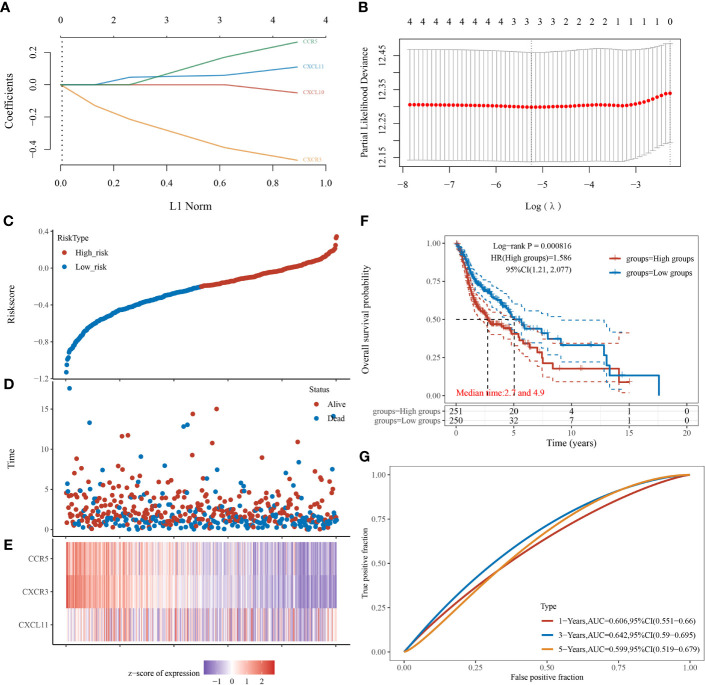
Establishment and verification of the risk-scoring system in patients with head-and-neck squamous cell carcinoma (*n* = 501). **(A)** LASSO coefficient profiles of CXCL10, CXCL11, CXCR3, and CCR5. **(B)** Partial likelihood deviance of variables revealed by LASSO-based Cox regression model. **(C)** Patients with HNSCC were divided into high- and low-risk groups (median as the cutoff) based on the risk scores. **(D)** Scatterplot of the survival status of patients with different risk scores. Abscissa represents risk score, and ordinate represents survival status. **(E)** Heatmap of expression levels of CXCL11, CXCR3, and CCR5 in tumors with different risk scores. **(F)** Kaplan–Meier analysis and log-rank test of patients with high- and low-risk scores. **(G)** Predictive accuracy of the risk-scoring system by time-dependent receiver operating characteristic curve (ROC) analysis.

CXCL11, CXCR3, and CCR5 were screened to be candidate genes related to prognosis of patients with HNSCC through LASSO Cox regression analysis (**Figures 4A, B** **)**. A risk-scoring system was then established based on the formula generated according to the expression of the three genes, which could calculate the risk scores of patients with HNSCC. The risk-scoring system was established as follows: risk score = (0.0636) * CXCL11 + (−0.4619) ;* CXCR3 + (0.2398) * ;CCR5. Patients with high-risk scores had significantly worse OS than low-risk patients (log-rank *p* < 0.001) (**Figures 4C–F**). The risk score was identified to be an independent risk factor for patients with HNSCC (HR, 1.586; 95% CI, 1.21~2.077). The prediction accuracy of the system had a good performance in predicting 1-year OS (AUC, 0.606; 95% CI, 0.551–0.66) and 3-year OS (AUC, 0.642; 95% CI, 0.59–0.695). In contrast, the system showed relatively poor performance in predicting 5-year OS (AUC, 0.599; 95% CI, 0.519–0.679) (**Figure 4G**).

### 3.5 Validation of Prognosis-Related Candidate Genes

**Figure 5:**
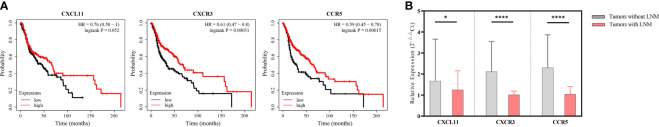
Identifying the prognostic role of CXCL11, CXCR3, and CCR5 in patients with head-and-neck squamous cell carcinoma (HNSCC). **(A)** Survival curves of HNSCC patients stratified by mRNA expression levels (median as the cutoff) (*n* = 501). **(B)** Comparing the relative expression levels of CXCL11, CXCR3, and CCR5 in tumors without lymphatic metastases (*n* = 18) and those with lymphatic metastases (*n* = 18) by unpaired Student’s *t*-test. Error bars represent the mean ± SEM. ^*^ *p* < 0.05; ns, *p* ≥ 0.05.

Of the three genes, CXCR3 had significant associations with OS of patients with HNSCC (HR, 0.64; 95% CI, 0.49–0.84; log-rank *p* = 0.001), so did CCR5 (HR, 0.76; 95% CI, 0.58–1; log-rank *p* = 0.048). Interestingly, patients with high expression of CXCL11 also had the tendency to live longer than those with low expression of CXCL11 (HR, 0.82; 95% CI, 0.62–1.07), but not statistically significant (log-rank *p* = 0.15) (**Figure 5A**). Also, the relative expression level of CXCL11 was significantly lower in LNM^+^ tumors compared with that in LNM^−^ tumors (*p* < 0.05), whereas CCR5 showed significant higher expression in LNM^+^ tumors compared with that in LNM^−^ tumors (*p* < 0.05). The relative expression level of CXCR3 in LNM^+^ tumors was mildly higher but not statistically significant (*p* = 0.436) (**Figure 5B**).

### 3.6 Ability of the Risk-Scoring System to Reflect the TIL Landscape

**Figure 6:**
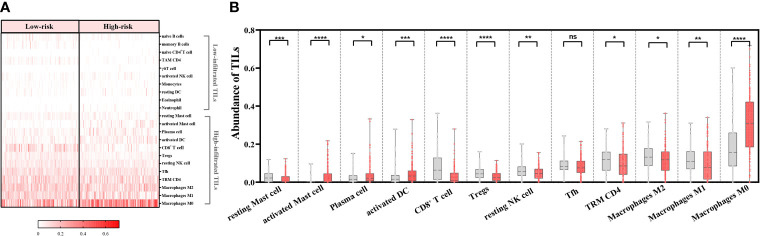
Ability of the risk-scoring system to characterize the tumor-infiltrating lymphocyte (TIL) landscapes in patients with head-and-neck squamous cell carcinoma (*n* = 439). **(A)** Heatmap of abundance of 22 TILs in the low- and high-risk groups. **(B)** Comparing the abundance of 12 TILs between the low- and high-risk groups by Mann–Whitney *U* test. ^****^ *p* < 0.0001; ^***^ *p* < 0.001; ^**^ *p* < 0.01; ^*^ *p* < 0.05; ns, *p* ≥ 0.05. Grey columns represent the low-risk group, and red columns represent the high-risk group.

Among the 22 TILs, M0, M1, and M2 macrophages had the highest infiltration rates (24.7%, 12.6%, and 10.8%, respectively), followed by resting memory CD4^+^ T cells (TRM CD4), follicular helper T cells (Tfh), CD8^+^ T cells, and resting NK cells. Ten subtypes of TILs (naïve B cells, memory B cells, naïve CD4^+^ T cells, TAM CD4, γδT cells, activated NK cells, monocytes, resting dendritic cells, eosinophils, and neutrophils) showed low abundance in both high- and low-risk patients (<5%) (**Figure 6A**). As for the remaining 12 types of TILs, we compared their abundance in high- and low-risk groups and found that 11 of them showed significant differences. Among these TIL subtypes, seven types of TILs (resting mast cells, CD8^+^ T cells, Tregs, resting NK cells, TRM CD4, M2 macrophages, and M1 macrophages) had significantly higher abundance in low-risk groups. In contrast, four types of TILs (activated mast cells, plasma cells, activated DCs, and M0 macrophages) had significantly higher abundance in high-risk groups. Tfhs showed no difference in the infiltration between the two groups (*p* > 0.05) (**Figure 6B**).

**Figure 7:**
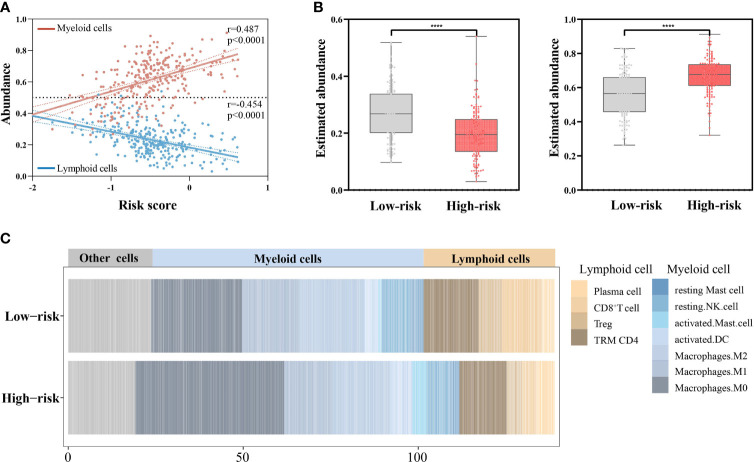
Associations of the risk scores with abundance of lymphoid cells and myeloid cells (*n* = 439). **(A)** Correlations of the risk scores with abundance of lymphoid cells and myeloid cells by Pearson’s correlation test. **(B)** Comparing the abundance of lymphoid cells (left) and myeloid cells (right) between the low- and high-risk groups by Mann–Whitney *U* test. ^****^ *p* < 0.0001. **(C)** Stacked column plots of abundance of TILs in low- and high-risk groups.

Intriguingly, with the increase in the risk scores, the overall abundance of lymphoid cells continuously decreased (*r* = −0.454, *p* < 0.0001) and that of myeloid cells increased (*r* = 0.487, *p* < 0.0001) (**Figure 7A**). Consistently, the lymphoid cells had greater infiltration in the low-risk groups (*p* < 0.0001) and the myeloid cells had greater infiltration in high-risk groups (*p* < 0.0001) (**Figures 7B, C** **)**.

### 3.7 Validating the Associations of the Risk Scores and Immune Checkpoints

**Figure 8:**
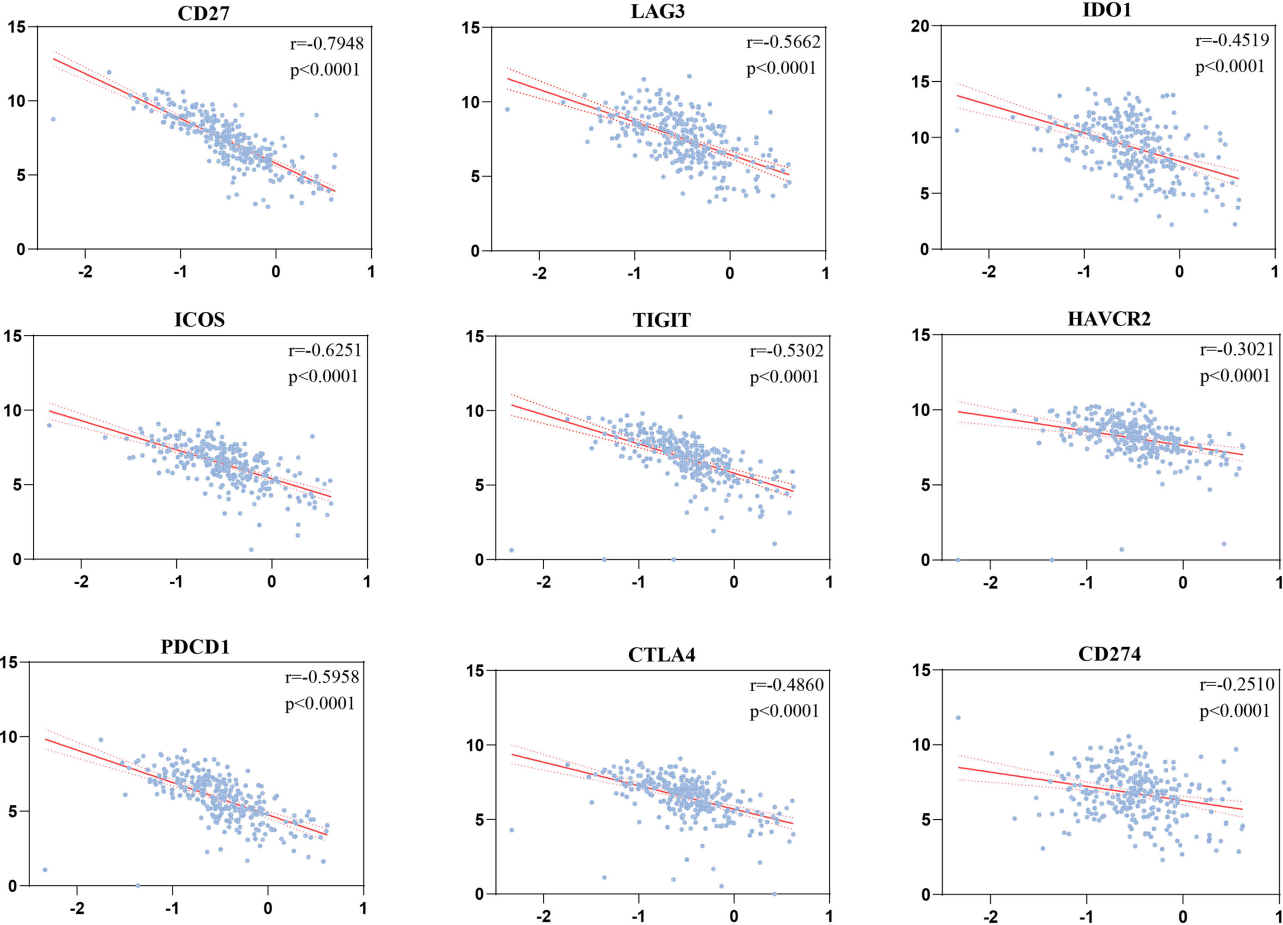
Correlations of the risk scores with immune checkpoints by Pearson’s correlation test (*n* = 501).

The risk scores were significantly correlated with CD27 (*r* = −0.7948, *p* < 0.0001), ICOS (*r* = −0.6251, *p* < 0.0001), PDCD1 (*r* = −0.5958, *p* < 0.0001), LAG3 (*r* = −0.5662, *p* < 0.0001), TIGIT (*r* = −0.5302, *p* < 0.0001), CTLA4 (*r* = −0.486, *p* < 0.0001), IDO1 (*r* = −0.4519, *p* < 0.0001), HAVCR2 (*r* = −0.3021, *p* < 0.0001), and CD274 (*r* = −0.251, *p* < 0.0001) (**Figure 8**).

### 3.8 Identifying the Prognostic Role of the Risk Scores

**Figure 9:**
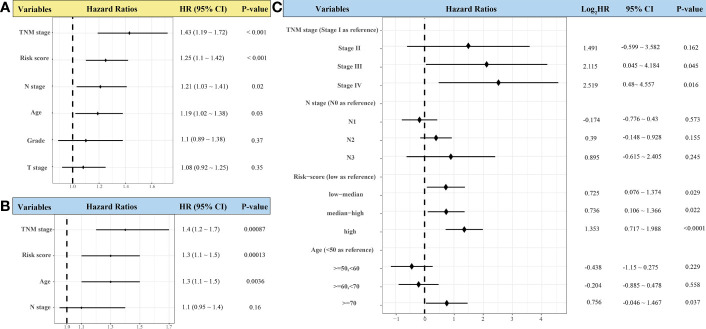
Forest plots showing univariate **(A)** and multivariate Cox regression analyses **(B, C)** of the effect of variables on overall survival of patients with head-and-neck squamous cell carcinoma (*n* = 439).

Risk score, TNM stage, and age were independent risk factors for OS of patients with HNSCC through univariate and multivariate Cox regression analyses (**Figures 9A, B**). Patients with low–medium-, median–high-, and high-risk scores had significantly higher mortality risks than those with low-risk scores (HR = 1.653, 1.666, and 2.554, respectively, *p* < 0.05). Patients at stage III and stage IV had significantly higher mortality risk than those at stage I (*p* < 0.05). The risk of death for patients aged >70 years was significantly higher than that of those aged <50 years (HR = 1.689; 95% CI, 1.032–2.764; *p* < 0.05) (**Figure 9C**).

### 3.9 Construction and Validation of a Nomogram Prediction Model

**Figure 10:**
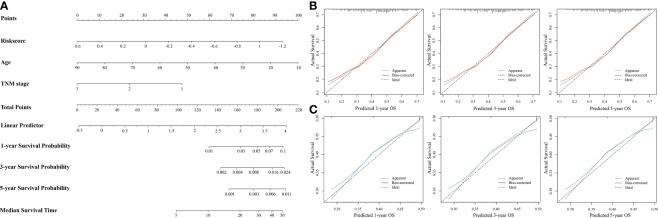
Establishment and evaluation of nomogram prediction model for overall survival (OS) of patients with head-and-neck squamous cell carcinoma (*n* = 439). **(A)** Nomogram based on independent risk factors for OS in patients with HNSCC. **(B)** Calibration curves of the nomogram model for 1-, 3-, and 5-year OS. **(C)** Calibration curves of TNM stage for 1-, 3-, and 5-year OS.

A nomogram was constructed to predict the OS of patients with HNSCC based on identified independent risk factors (risk score, TNM stage, and age) (**Figure 10A**). The prediction model displayed better discrimination ability than the TNM stage for predicting OS (c-index = 0.64 vs. 0.57, respectively). The calibration curves for probability of 1-, 3-, and 5-year OS showed good agreement between nomogram prediction and actual observation, which also performed better than the TNM stage (**Figures 10B, C** **)**.

## 4 Discussion

Although the associations between TILs and cancer outcomes vary according to cell specificity and tumor heterogeneity, pan-cancer analysis had revealed that higher estimated T-cell fractions are generally correlated with superior survival (37). Massive evidence supports the antitumor role of CD3^+^ T and CD8^+^ T cells in colorectal cancer (38), breast cancer (39), and nonsmall cell lung cancer (40). Strong infiltration of CD8^+^ T cells has been generally associated with a favorable prognosis of patients with cancer (41–43). Moreover, immunotherapies are mainly aimed to reinvigorate antitumor immunity mediated by CD8^+^ cytotoxic T lymphocytes (CTLs) (23). CD4^+^ T cells can eliminate tumor cells by promoting the functions of CTLs or modulating the TME (44, 45). Greater infiltrations of CD4^+^ and CD8^+^ T lymphocytes have been identified to be associated with improved OS for HNSCC (46, 47). Consistently, we found higher CD8^+^ and CD4^+^ T-cell infiltration in HNSCC tumor tissues than adjacent normal tissues (*p* < 0.05) in our cohort. The density of CD8 and CD4 was highly correlated (*r* = 0.256, *p* < 0.05), indicating their synergy in the TME. The prognostic role of tumor-infiltrating FoxP3^+^ T lymphocytes in patients with HNSCC is controversial. Boxberg et al. reported that patients with HNSCC with lower density of Foxp3^+^ T lymphocytes tended to have worse OS and disease-free survival (48). In contrast, Mehtap et al. reported that FoxP3 was correlated with advanced tumor stages and poor prognosis (49). A pan-cancer meta-analysis revealed the heterogeneity of the prognostic roles of FoxP3s among tumor sites and the antitumor role of FoxP3 in HNSCC (OR, 0.69; 95% CI, 0.50~0.95; *p* < 0.05). It is hypothesized that the positive effect of FoxP3^+^ Tregs may be partially attributed to its ability to suppress inflammatory response, which may promote tumor progression (50, 51).

In this study, we found that both innate and adaptive immune responses engaged in the lymphatic metastatic process of HNSCC tumors, which reminded us the importance of investigating TILs. Among tumor-infiltrating T lymphocytes, CD8^+^ T cells seemed to actively participate in the antitumor LNM response in patients with HNSCC, which was also identified through the Chi-squared analysis of the IHC scores of CD8 (*p* = 0.001). Considering the cytotoxic effect of CD8^+^ T cells and regulation function of CD4^+^ T cells and Tregs in tumor immunity, chemokines closely related to the three TIL subtypes can reflect tumor immune environment to some degree. The role of CXCL11 in tumor immunity is controversial. Notably, CXCL11 can promote antitumor immunity to benefit survival, as in patients with colon adenocarcinoma (52). However, CXCL11 is a potential antagonist of CXCL10 and CXCL11 because of its higher affinity for CXCR3 (53). CXCL11 also binds to CXCR7, implicating it in tumor invasiveness (54). The mechanism underlying the function of CXCL11 in the tumor environment may explain its negative association with prognosis. Additionally, the role of CCR5 in HNSCC tumor immunity is also intriguing. In our study, we found that patients with HNSCC with higher CCR5 expression had significantly better OS (HR, 0.59; 95% CI, 0.45–0.78). However, CCR5 contributes to negative effect (coefficient, 0.2398) on survival rates in LASSO regression model. Also, CCR5 had significantly higher expression in tumors with LNM than in those without LNM (*p* < 0.05) in our cohort. Some studies reported that greater cytoplasmic CCR5 expression is correlated with a poor prognosis of patients with cancer because it induces cancer hallmarks (55), cancer homing to metastatic sites (56), and tumor invasion (57). A few studies also focused on the tumor-promoting role of CCR5 in HNSCC (58–60), which are consistent with our partial findings. Thus, CCR5 may not be an independent prognostic factor for outcome of patients with HNSCC, and further investigation is warranted.

In contrast to the immune surveillance role of lymphocytes in the TME, myeloid cells may promote tumor growth and metastasis through by favoring the TME (61–63). Zhang et al. implicated the role of CD8^+^ T cells in attenuating the protumor activity of myeloid cells in the premetastatic TME by compromising Stat3, which indicated its therapeutic potential (64). Consistently, the competition for between lymphoid cells and myeloid cells in TME of HNSCC were also presented in our study. Tumors with higher risk scores tended to be infiltrated by greater abundance of myeloid cells and less abundance of lymphoid cells, and had worse prognosis, compared with those with lower risk scores. Although, many studies have assessed the abundance of individual lymphocytes or myeloid cells in tumor tissues to predict the prognosis of patients and potential sensitivity to adjuvant chemotherapy and immunotherapy (12). However, the tumor immune microenvironment is an intricated assembly of varieties of TILs. They interact to shape the TME that may be antitumor or tumor promoting. Based on this, the risk-scoring system we established in the study comprehensively evaluates the infiltrations of various significant TILs and provides a more reliable theoretical basis for stratifying patients receiving immune therapies.

This study had some limitations. First, the method used to quantify the density of IHC markers may not fully reflect *in vivo* expression patterns. Second, the performance of the risk scores in predicting OS of patients with HNSCC was unsatisfactory. We speculate that it may be because the risk-scoring system only includes genes closely relevant to the tumor immune microenvironment. However, the TME not only consists of immune cells but also fibroblasts, endothelial cells, normal epithelial cells, nutrients, etc. These admixtures have been extensively researched and thought to involve in the tumor growth (65). Additionally, the prognosis or tumor progression is mainly regulated by tumor cells. Therefore, more genes associated with tumor progression should be incorporated into the system to improve the prediction accuracy of prognosis of patients with HNSCC. Third, the discrimination of constructed nomogram was limited (c-index, 0.64; 95% CI, 0.55–0.73), despite its better performance than TNM stage. We speculate that it may be because the risk score only evaluates the patient’s prognosis from the perspective of tumor LNM. However, tumor progression is a complicated process in which tumor cells interact with TME for mutual promotion. Therefore, some indicators related to the invasive ability of tumor cells and identified to be independent risk factors (e.g., HPV status, depth of invasion, extranodal extension) should also be included in the prediction model to improve the reliability, which were not assessed in this study due to the retrospective nature of the data (66–68). There is still room for improvement of the nomogram in the prognosis prediction of HNSCC. Prospective cohort studies involving a large number of patients are needed to improve it in the future.

## 5 Conclusion

We identified the involvement of CD8^+^ T cells in antitumor immunity during the process of tumor lymphatic metastasis and established an immune-feature-based three-gene-signature risk-scoring system to predict HNSCC prognosis. The risk-scoring system had good performance in characterizing the immune landscape in HNSCC and might benefit clinical patient risk stratification. The constructed nomogram could be a robust supplement to the TNM stage in the prediction of clinical prognoses. Further demonstrations of their prediction values in the clinical level are needed in the future.

## Data Availability Statement

The sequencing data from this study are publicly available under GEO series number GSE176221 through the NCBI Gene Expression Omnibus (GEO) (http://www.ncbi.nlm.nih.gov/geo) (69).

## Ethics Statement

The studies involving human participants were reviewed and approved by the Ethics Committee of the First Affiliated Hospital, College of Medicine, Zhejiang University. The patients/participants provided their written informed consent to participate in this study. Written informed consent was obtained from the individual(s) for the publication of any potentially identifiable images or data included in this article.

## Author Contributions

HZ and YZ initiated, designed, and coordinated the study. YZ performed data acquisition, analysis, and interpretation. XL performed prediction model establishment. JY provided clinical information and performed the prognostic validation of the candidate genes. KQ collected tissues. All authors contributed to the article and approved the submitted version.

## Conflict of Interest

The authors declare that the research was conducted in the absence of any commercial or financial relationships that could be construed as a potential conflict of interest.

## Publisher’s Note

All claims expressed in this article are solely those of the authors and do not necessarily represent those of their affiliated organizations, or those of the publisher, the editors and the reviewers. Any product that may be evaluated in this article, or claim that may be made by its manufacturer, is not guaranteed or endorsed by the publisher.
